# Supplementary material for: Chemokine-Decorated Nanoparticles Target Specific Subpopulations of Primary Blood Mononuclear Leukocytes
Source: Nanomaterials (Basel). 2022 Oct 11;12(20):3560. doi: 10.3390/nano12203560 (PMC9609899; doi:10.3390/nano12203560)
Supplement: Supplementary file 1 [file nanomaterials-12-03560-s001.zip › nanomaterials-1938700-supplementary.pdf]

nanomaterials

Supplementary Materials

Chemokine-Decorated Nanoparticles Target Specific Subpopulations of Primary Blood Mononuclear Leukocytes

Anissa Pisani [1,2], Roberto Donno [3], Giulio Valenti [3], Pier Paolo Pompa [1], Nicola Tirelli [3,*] and Giuseppe Bardi [1,*]

[1] Nanobiointeractions & Nanodiagnostics, Istituto Italiano di Tecnologia, via Morego 30, 16163 Genova, Italy
[2] Department of Chemistry and Industrial Chemistry, University of Genova, via Dodecaneso 31, 16146 Genova, Italy
[3] Laboratory of Polymers and Biomaterials, Istituto Italiano di Tecnologia, 16163 Genova, Italy
* Correspondence: nicola.tirelli@iit.it (N.T.); giuseppe.bardi@iit.it (G.B.); Tel.: +39-010-289-6923 (N.T.); +39-010-289-6519 (G.B.)

Scheme of the Project

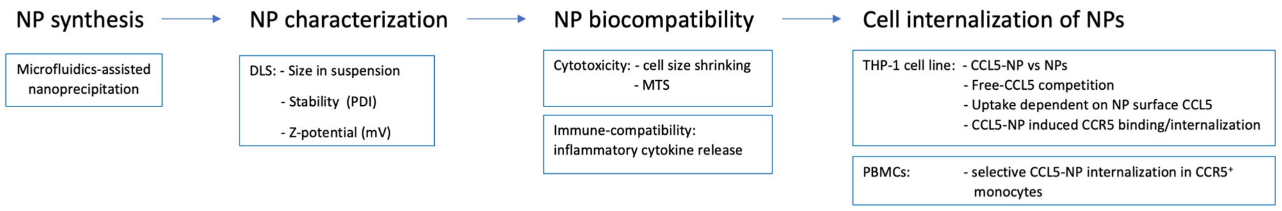

Nanoparticle Preparation and Characterization

Materials

Poly(D,L-lactide-co-glycolide) RG502 Resomer (PLGA, acid terminated PLGA-COOH), Pluronic F127, biotin, cysteamine hydrochloride, 2-mercaptoethanol, sodium azide, d6-dimethylsulfoxide (d6-DMSO), and Spectra/Por® dialysis membranes (MWCO 3.5 kDa) were purchased from Sigma Aldrich (Merk Life Science, Milan, Italy). HEPES sodium salt and 2 M hydrochloric acid solution were supplied from Alfa Aesar (Thermo Fisher, Kandel, Germany). The 4-(4,6-dimethoxy-1,3,5-triazin-2-yl)-4-methylmorpholinium chloride (DMTMM) was from Fluorochem (Hadfield, UK).

Preparation of Pluronic F127-Biotin (F127-BIO)

Pluronic F127-α,ω-bis(vinyl sulfone) (F127-VS) with 63% molar percentage of OH groups converted into vinyl sulfone moieties was prepared as previously reported [237]. In total, 100 mg of F127-VS (corresponding to 10 μmol of vinyl sulfone groups) were dissolved in 25 mL of 50 mM HEPES buffer at pH 7.4 and sonicated for 10 min to degas. Then, 1.4 mg (12 μmol, 1.2 eq.) of cysteamine hydrochloride was added and the solution was left overnight on an orbital shaker. Afterwards, in a second reaction vessel, 7 mg of biotin (30 μmol, 2.5 eq.) dissolved in 100 μL of DMSO was mixed for 5 min with 12 mg of DMTMM (45 μmol, 3.8 eq.) dissolved in 200 μL of HEPES buffer. DMTMM and biotin were then added to the modified Pluronic. After 24 h a large excess of 2-mercaptoethanol (1.5 mL) was added to quench all the remaining vinyl sulfone groups and the reaction was allowed to proceed overnight. The product was finally purified by dialysis against deionized water (MWCO 3.5 kDa) and recovered after freeze drying with 80% mass recovery. Quantitative conversion of the double bond was confirmed by 1H NMR. Specifically, 87% (molar percentage) of the vinyl sulfone groups were biotinylated, while the remaining groups were quenched with 2-mercaptoethanol. 1H NMR was recorded on 1 wt.% polymer solutions in d6-dimethylsulfoxide (d6-DMSO) using a Bruker AVANCE III 400 MHz spectrometer equipped with a Broad Band Inverse probe. The residual solvent signal was used as internal reference (ppm). Pluronic F127-biotin (F127-BIO): d6-DMSO, δ (ppm): 0.81–0.92 (m, H-h), 1.04 (d, J = 8 Hz, methyl group in propylene oxide units), 1.15–1.40 (m, H-g), 1.43–1.55 (m, H-i), 2.57–2.65 (m, H-k), 2.80–2.85 (m, H-e), 3.48–3.54 (m, methylene and methine groups in both ethylene and propylene units), 4.12–4.16 (m, H-d, d'), 6.34 (bs, H-c), 6.39 (bs, H-c').

Nanoparticle Preparation and Decoration

The automated microfluidic Asia 320 system (Syrris, Royston, UK) was used for all preparations. A 0.015 wt.% surfactant aqueous solution (25% Pluronic F127-VS and 75% Pluronic F127) was mixed with a 0.31 wt.% PLGA acetone solution in an Asia reaction chip (26 μL Micromixer chip, Syrris, Royston, UK). A schematic view of the process in SI Figure 1. The flow rates were controlled to have an acetone/water flow rate ratio of 0.2 and a total flow of 2 mL/min. The collected nanoparticle (NP) suspension was left at 30 °C for 12 h under stirring in order to evaporate the acetone. MilliQ water was then added in amounts equal to the volume loss, to maintain the initial nanoparticle concentration (i.e., 0.052 wt.%). The NPs suspension was then sterilized by filtering it with 0.22 μm cellulose acetate filters. The streptavidin-mediated decoration of PLGA-NPs involved two steps: firstly, streptavidin was mixed with 3 equivalents of biotin-containing molecules in order to occupy three of the four available binding sites; secondly, the aforementioned mixture was added to the biotinylated NPs with a 2:1 molar ratio between the biotin bound on the NPs and the streptavidin. Specifically:

Control NPs—10 μL of a 100 μM solution of Atto 610-biotin (Sigma-Aldrich, Saint Louis, MO, USA) in DMSO (equivalent to 1 nmol) was mixed with 290 μL of 7 μM solution of biotin in MilliQ water (equivalent to 2 nmol), followed by 50 μL of streptavidin reconstituted at 1 mg/mL, corresponding to 1 nmol of protein units. The procedure was

allowed to proceed for 15 min. The mixture was then added to 650 µL of the 0.052 wt.% nanoparticle dispersion, vortexed for 10 s and allowed to rest for 15 min.

CCL5-decorated NPs—10 µL of a 100 µM solution of Atto 610-biotin in DMSO (equivalent to 1 nmol) was mixed with 280, 270, 250, 210 and 190 µL of 7 µM solution of biotin in MilliQ water (equivalent to 1.96, 1.86, 1.66, 1.26, 1.06 nmol, respectively), and 10, 20, 40, 80 and 100 µL of a 10 µM solution of biotinylated human CCL5 in MilliQ water (equivalent to 0.1, 0.2, 0.4, 0.8, 1 nmol, respectively) (Chemotactics, San Diego, CA, USA), followed by 50 µL of streptavidin (Prospec, Rehovot, Israel) reconstituted at 1 mg/mL, corresponding to 1 nmol of protein units. The procedure was allowed to proceed for 15 min. The mixture was then added to 650 µL of the 0.052 wt.% nanoparticle dispersion, vortexed for 10 s and allowed to rest for 15 min.

Biocompatibility of CCL5 Functionalized Nanoparticles

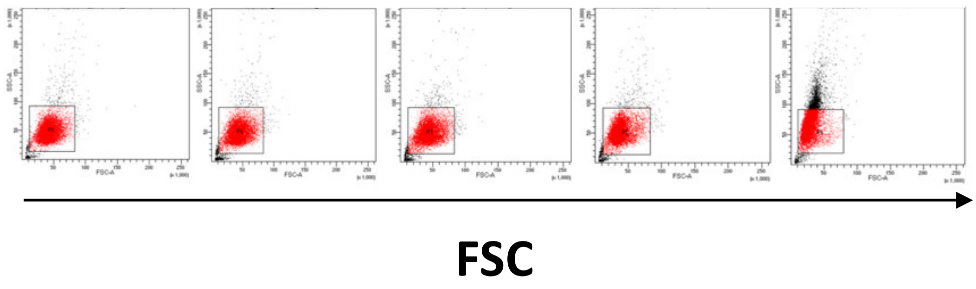

(**a**)

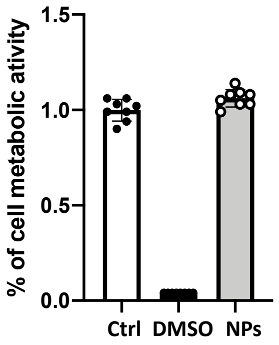

(**b**)

Figure S1. (**A**) Flow cytometry dot plot graphs of THP-1 cells treated with different concentration of streptavidin-conjugated NPs ranging from 32.5 µg/mL to 325 µg/mL. Forward Scattering (FSC) parameter on X-axis displays the relative cell size, whereas Side Scattering (SSC) on the Y-axis shows the granularity of the cells. (**B**) MTS measured percentage of metabolic activity (left graph) of THP-1 in 5% Human Serum (HS) supplemented medium. Data are represented in percentage relative to untreated control. Untreated THP-1 cells (Ctrl, white bar), THP-1 cells treated with streptavidin conjugated-NPs (grey bar). 10% DMSO (black bar) was used as positive control.

Methods

Flow cytometry and MTS methods and materials can be found in the main text.

CCL5-NPs Reduced Internalization by Free CCL5 Competition

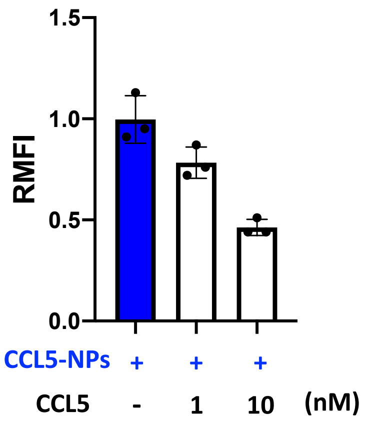

Figure S2. Internalization of 65 µg/mL CCL5-NPs in THP-1 cells pre-treated or not with CCL5. Bars represent the relative median fluorescence intensity (RMFI) of at least three independent experiments +/− SD (error bars).

Method

THP-1 cells (2 × 10^5 cells/ml) were collected and samples to desensitize were incubated for 2h at 37 °C with 1 and 10 nM human CCL5 (Chemotactics, San Diego, CA, USA) before adding non-functionalized NPs and CCL5-NPs for 45min at 37 °C. Then, cells were washed twice at 4 °C. The resulting median fluorescence intensity was analyzed by flow cytometry with BD FACSDiva software 6.0 provided by BD Biosciences (San Jose, CA, USA), gating living cells based on FSC and SSC. In total, 3 × 10^4 events per sample were acquired.

CCR5 Expression on Primary Monocytes

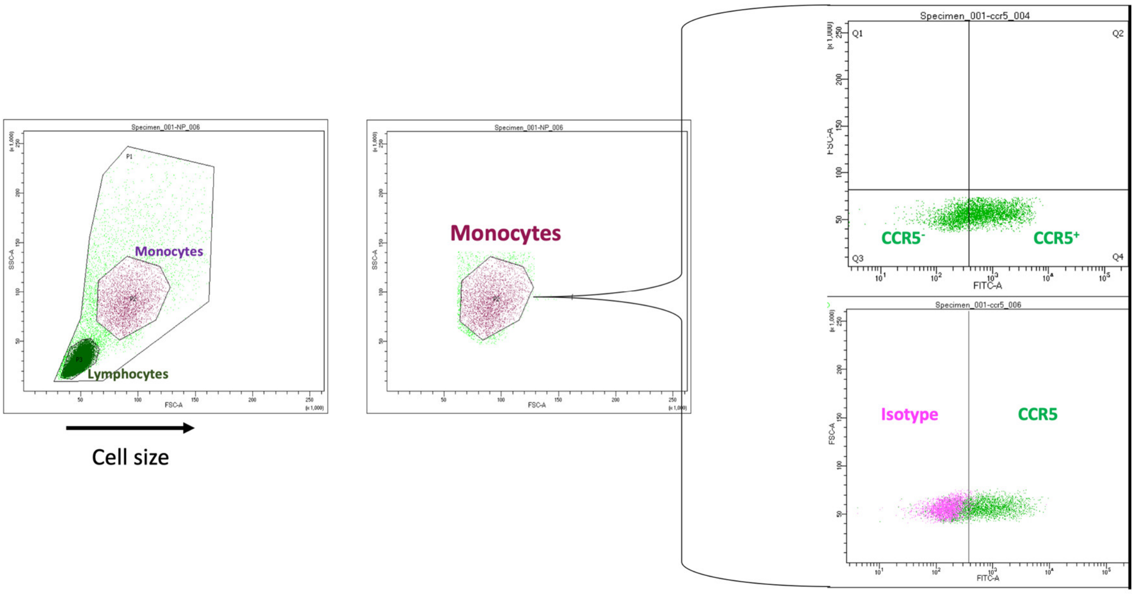

Figure S3. Plot obtained by flowcytometry representing human PBMCs. Lymphocytes are coloured in dark green and monocytes in dark pink. Inset: (Right upper plot) Monocytes stained with anti-CCR5 antibody. In Q3 is present the subpopulation of cells $CCR5^-$ whereas in Q4 there is the subpopulation of cells $CCR5^+$. (Right lower plot) Monocytes stained with the isotype in pink and the anti-CCR5 antibody in green.

Method

PBMCs (2×10^5 cells/mL) were centrifuged at 300× *g* for 5 min, washed twice, resuspended in serum-free medium supplemented with 0.5% BSA (Miltenyi Biotec, Bergish, Germany) and incubated for 30 min at 37 °C for starvation. Then cells were incubated with fluorescently labeled antibodies (Vio® Bright B515 anti-human CCR5 (Miltenyi Biotec, Bergish, Germany); Vio® Bright B515 REA Control Antibody (S), human IgG1 (Miltenyi Biotec, Bergish, Germany)) at the manufacturer's recommended concentration for 30 min on ice in the dark, then washed twice and resuspended in RPMI 1640 without phenol red (Thermo Fisher Scientific, Waltham, MA, USA) at 4 °C.

Cell-associated fluorescence was measured by flow cytometry with BD FACSDiva software 6.0 provided by BD Biosciences (San Jose, CA, USA), gating living cells based on FSC and SSC. In total, 5×10^4 events per sample were acquired.
